# Serum levels of the C-terminal pro-peptide of type V collagen have prognostic potential and are associated with a normal subtype of desmoplasia in a retrospective cohort of patients with esophagogastric cancer

**DOI:** 10.1177/17588359261459109

**Published:** 2026-06-28

**Authors:** Benthe H. Doeve, Neel I. Nissen, Vera Weeda, Mark P. G. Dings, Sybren L. Meijer, Hanneke W. M. van Laarhoven, Maarten F. Bijlsma, Nicholas Willumsen

**Affiliations:** Department of Medical Oncology, Amsterdam UMC, University of Amsterdam, Amsterdam, the Netherlands; Imaging and Biomarkers, Cancer Center Amsterdam, Amsterdam, the Netherlands; Laboratory for Experimental Oncology and Radiobiology, Center for Experimental and Molecular Medicine, Amsterdam UMC, University of Amsterdam, Amsterdam, the Netherlands; Biomarkers & Research, Nordic Bioscience, Herlev, Denmark; Department of Medical Oncology, Amsterdam UMC, University of Amsterdam, Amsterdam, the Netherlands; Imaging and Biomarkers, Cancer Center Amsterdam, Amsterdam, the Netherlands; Laboratory for Experimental Oncology and Radiobiology, Center for Experimental and Molecular Medicine, Amsterdam UMC, University of Amsterdam, Amsterdam, the Netherlands; Imaging and Biomarkers, Cancer Center Amsterdam, Amsterdam, the Netherlands; Laboratory for Experimental Oncology and Radiobiology, Center for Experimental and Molecular Medicine, Amsterdam UMC, University of Amsterdam, Amsterdam, the Netherlands; Imaging and Biomarkers, Cancer Center Amsterdam, Amsterdam, the Netherlands; Department of Pathology, Amsterdam UMC, University of Amsterdam, Amsterdam, the Netherlands; Department of Medical Oncology, Amsterdam UMC, University of Amsterdam, Amsterdam, the Netherlands; Imaging and Biomarkers, Cancer Center Amsterdam, Amsterdam, the Netherlands; Laboratory of Experimental Oncology and Radiobiology, Amsterdam UMC, University of Amsterdam, RDC Adore ZH 3J079, Van der Boechorststraat 6b, Amsterdam 1081BT, The Netherlands; Imaging and Biomarkers, Cancer Center Amsterdam, Amsterdam, the Netherlands; Laboratory for Experimental Oncology and Radiobiology, Center for Experimental and Molecular Medicine, Amsterdam UMC, University of Amsterdam, Amsterdam, the Netherlands; Oncode Institute, Amsterdam, the Netherlands; Biomarkers & Research, Nordic Bioscience, Herlev, Denmark

**Keywords:** collagen V, desmoplasia, esophagogastric cancer, normal subtype, stroma biomarkers

## Abstract

**Background:**

The role of stroma in esophagogastric cancer is ambiguous, with both tumor-promoting and tumor-restrictive properties. Several non-invasive biomarkers of stroma have recently been identified, but their relationship with stromal composition and clinical outcome in esophagogastric cancer remains insufficiently understood.

**Objectives:**

We aimed to investigate the association of serum stromal biomarkers with overall survival and stroma composition in esophagogastric cancers.

**Design:**

Eleven stromal biomarkers were retrospectively measured in pre-treatment serum of 78 patients with esophagogastric cancer and 11 healthy controls.

**Methods:**

Elastic net Cox regression was applied to associate serum concentration of stromal biomarkers with overall survival in patients with esophagogastric cancer. These associations were later confirmed with Kaplan–Meier curves and multivariate Cox regression analysis. RNA-sequencing was performed on 17 matched tumor biopsies and an independent validation cohort of 100 pre-treatment biopsies to explore the relation between stromal biomarker and stromal subtype.

**Results:**

This study revealed that serum formation fragment of collagen V as a stromal biomarker was high in patients with esophagogastric cancer compared to healthy subjects. A high serum concentration of the formation fragment of collagen V was independently prognostically favorable in patients with esophagogastric cancer. The formation of collagen V correlated with a distinct normal stroma subtype in the matched tumor biopsies. A gene signature specific for the formation fragment of collagen V was also independently prognostically favorable and correlated with a normal stroma subtype in the validation cohort.

**Conclusion:**

In conclusion, the formation of collagen V reflects a distinct normal stroma subtype in patients with esophagogastric cancer that associates with favorable outcomes.

## Plain language summary

A collagen-related blood marker may help predict survival and is linked to differences in the tissue surrounding tumors in esophagogastric cancer

Why was the study done? Esophagogastric cancer (cancer of the esophagus and/or stomach) does not consist of cancer cells alone. Tumors also contain stroma, the supportive tissue around the cancer made up of collagens and other structural proteins. Stroma can sometimes help tumors grow, but in other cases it may restrict them. This study looked at whether stroma-related markers in blood are linked to different stroma types in esophagogastric tumors, and if that informs on prognosis. What did the researchers do? The researchers measured 11 stroma-related biomarkers in blood (serum) taken before treatment from 78 people with esophagogastric cancer, and 11 healthy people for comparison. They then used a statistical modeling method to see which biomarkers were linked to overall survival. After that, they examined 17 tumor biopsy samples from the same patients to understand whether the most important biomarker was connected to a specific subtype of stroma. They validated their findings in a different group of 100 patients for which they had gene expression data available. What did the researchers find? A fragment in the blood indicating formation of collagen V was higher in patients with esophagogastric cancer compared to healthy people. In patients, higher levels of this marker were linked to better survival. In the biopsies, this marker was associated with a distinct “normal stroma” subtype. In the larger group of patients, high expression of genes related to this marker were also linked to better survival and a “normal stroma” subtype. What do the findings mean? Formation of collagen V in the surrounding tissue of esophagogastric tumors indicates a more “normal like” stroma and this is linked to better outcomes for patients

## Background

Cancers of the esophagus, esophagogastric junction, and stomach, together also known as esophagogastric cancer (EGC), accounted for 13% of cancer-related deaths worldwide in 2020.^1^ Their partial overlap is illustrated by molecular subtyping, and they are managed with similar perioperative and advanced disease treatment.^2–4^ In recent years, treatment advances with immune checkpoint inhibitors^5–7^ and targeted agents^8^ have demonstrated survival benefit in selected patient populations. These studies selected patients based on human epidermal growth factor receptor 2 (HER2) amplification, programmed death-ligand 1 (PD-L1) expression^6,9,10^, and histological features such as complete pathological response and radical resection.^7^ This underscores the increasing importance of biomarkers for patient stratification in clinical practice.

The stroma of these tumors consists of non-tumor cells such as fibroblasts and immune cells, embedded in a continuously remodeled protein network called the extracellular matrix (ECM). The main proteins in the ECM are collagens, and their turnover is tightly regulated by metalloproteinases (MMPs) and inhibitory enzymes. Cancer cells dysregulate these dynamics by producing MMPs and by turning fibroblasts into cancer-associated fibroblasts (CAFs), which then excessively produce multiple collagen types.^11–13^ This desmoplastic stroma can act as a physical barrier protecting the tumor from therapies, but also promotes tumor cell proliferation, stemness, and epithelial-to-mesenchymal transition.^14–18^ Subsequently, desmoplasia has been associated with poorer overall survival and is predictive of treatment response in several cancer types.^19^ In EGC, the tumor-stroma ratio and upregulated expression of MMPs have been reported to be associated with poor survival, increased lymph node metastases, and poorer pathological response to treatment.^20–24^ In contrast, tumor stroma has been shown to have tumor-restrictive properties as well, for instance by acting as a steric obstacle for cancer cell motility.^11,26^ This ambivalent nature is not as well studied in EGC as it has been in pancreatic ductal adenocarcinoma (PDAC),^11,25–27^ where distinct stromal subtypes have been shown to differentially associate with prognosis.^28^

The degradation and formation fragments associated with increased collagen turnover and increased MMP production in desmoplasia are released in the bloodstream and can be used as biomarkers. Serum levels of fragments of collagen I, III, IV, V, and VI can distinguish healthy controls from patients with melanoma, PDAC, biliary tract, lung, colon, breast, and ovarian cancer.^29–35^ Collagen III fragments in the serum are predictive of treatment response, progression-free survival, and overall survival in patients with melanoma, colorectal cancer, biliary tract cancer, and PDAC.^32,35–39^ Similarly, fragments of collagen type VI have been prognostic in patients with colorectal and biliary tract cancer, whereas fragments of collagen V and XI have been prognostic in patients with PDAC.^34,35,38,40^ Despite their reported relevance in other solid tumors, the prognostic significance of serum levels of collagen formation and degradation fragments in EGC remains unknown. In addition, their relationship with the stroma of the tumor microenvironment has not been defined. Therefore, we investigated the associations between serum stromal biomarkers, overall survival, and tumor-stroma composition in patients with EGC.

## Methods

### Patients and patient material collection

**Figure 1.**
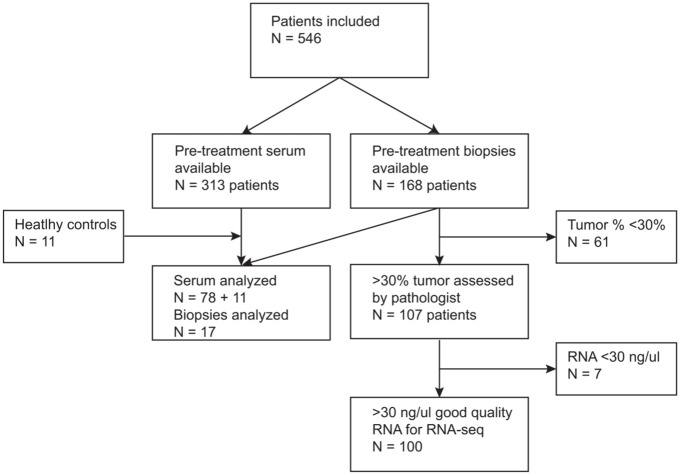
Sample flow of included patient samples.

This study was conducted in accordance with the Declaration of Helsinki and international standards of good clinical practice. Patients were recruited from the Amsterdam UMC between January 2015 and January 2018 and provided written informed consent (BiOES; METC 2013_241). Eligible patients had pathologically confirmed EGC of any stage and were 18 years or older. Patient material was collected before, during, and after treatment during clinically indicated blood withdrawals or interventions to collect tumor tissue for diagnostic purposes. Serum was obtained by centrifugation of blood according to the biobank-specific protocol, and tumor biopsies were snap frozen in liquid nitrogen. All samples were stored at −80°C until analysis. We budgeted the analysis of 78 baseline samples and 11 healthy controls. The selection of samples was based on the availability of other translational data. Matched tumor biopsies of the primary tumor (*n* = 13) or metastatic site (*n* = 4) were available for 17 patients. We also collected a biopsy cohort of non-matched untreated primary tumor biopsies (*n* = 100). Sample flow is depicted in Figure 1, and baseline characteristics are listed in Tables 1 and 2.

**Table 1.** Clinical characteristics of patients included in baseline serum analysis.

Clinical variable	Study population (*n* = 78)
Age (years)	
Median (IQR)	62 (57–68)
Sex	
Female	16 (20.5%)
Male	62 (79.5%)
cT-stage	
T1	2 (2.6%)
T2	13 (16.7%)
T3	55 (70.25)
T4	3 (3.8%)
Tx	5 (6.4%)
cN-stage	
N0	20 (25.6%)
N1	28 (35.9%)
N2	18 (23.1%)
N3	9 (11.5%)
Nx	3 (3.8%)
cM-stage	
M0	40 (51.3%)
M1	35 (44.9%)
Mx	3 (3.8%)
Location	
Esophagus	60 (76.9%)
Gastro-esophageal junction	13 (16.7%)
Stomach	5 (6.4%)
Histology	
Adenocarcinoma	57 (73.2)
Squamous cell carcinoma	20 (26.0)
Other	1 (0.8)
Treatment type	
Chemoradiotherapy with or without surgery	41 (52.6%)
Fluoropyrimidine/platinum-based systemic therapy only	37 (47.4%)

Percentages do not add to 100% due to rounding.

**Table 2.** Clinical characteristics of validation cohort (GSE254942).

Clinical variable	Study population (*n* = 100)
Age (years)	
Median (IQR)	66 (58–73)
Sex	
Female	19 (19%)
Male	81 (81%)
cT-stage	
T1	0 (0%)
T2	23 (23%)
T3	68 (68%)
T4	7 (7%)
Tx	2 (2%)
cN-stage	
N0	19 (19%)
N1	50 (50%)
N2	27 (27%)
N3	3 (3 %)
Nx	1 (1 %)
cM-stage	
M0	87 (69.2%)
M1	12 (26.9%)
Mx	1 (3.8%)
Location	
Esophagus	71 (71%)
Gastro-esophageal junction	23 (23%)
Stomach	4 (4%)
Unknown	2 (2%)
Histology	
Adenocarcinoma	81 (81%)
Squamous cell carcinoma	16 (16%)
Other	3 (3.0%)
Treatment type	
Chemoradiotherapy with or without surgery	74 (74%)
Fluoropyrimidine/platinum-based systemic therapy only	8 (8%)
Fluoropyrimidine/platinum/taxane with or without surgery	9 (9%)
Unknown	9 (9%)

Percentages may not sum to 100% due to rounding.

### Tumor biopsy processing and RNA-sequencing

Snap-frozen biopsies were cut into 20 µm slices. An experienced pathologist (SLM) determined the tumor percentage of each biopsy in an H&E-stained midsection of 5 µm. Tumor percentage was at least 30% in all biopsies. We isolated total RNA according to the manufacturer’s protocol with the AllPrep DNA/RNA/miRNA universal kit (Qiagen, Hilden, Germany). Samples were sent for RNA-sequencing if they met a threshold of 20 ng/µL on the Nanodrop (Thermo Fisher, Waltham, MA). Library preparation was performed using the total library prep RiboErase (Roche, Basel, Switzerland). Samples were sequenced on the Illumina HiSeq4000 with single 50bp reads at 100 million reads per sample. The data were of high quality according to FastQC42. Reads were aligned to the human reference genome (NCBI37/hg19) using STAR v2.7.1 and annotated with Gencode v32, retaining only uniquely mapped reads. Data were uploaded and analyzed in the R2 Genomics Analysis and Visualization Platform. Gene expression data supporting the conclusions of this article are accessible at GEO under GSE254942, and on Figshare: https://figshare.com/s/2cf7cd28805715224594.

### Biomarker measurements

We measured serum concentration of eleven stromal biomarkers using enzyme-linked immunosorbent assay (ELISA) and electro-chemiluminescence (ECLIA)-based assay. The ELISAs measured the neo-epitope of MMP-9 mediated degradation of type III collagen (C3M),^41^ the neo-epitope of MMP-2 and MMP-9 mediated degradation of type V collagen (C5M),^42^ the neo-epitope of MMP-2 mediated degradation of type VI collagen (C6M),^43^ the released N-terminal pro-peptide of type III collagen (PRO-C3),^44^ the released C-terminal pro-peptide of type V collagen (PRO-C5),^45^ the C-terminal of released C5 domain of type VI collagen α3 chain (PRO-C6),^43^ the released N-terminal pro-peptide of type XI collagen cleaved at amino acid A’511↓ (PRO-C11-511),^40^ the C-terminal of type XIX collagen (PRO-C19),^46^ the C-terminal of type XXVIII collagen (PRO-C28),^47^ the neo-epitope of MMP-2 and MMP-8 mediated degradation of Vimentin (VIM).^48^ The ECLIA-based assay measured the neo-epitope of granzyme B-mediated degradation of type IV collagen (C4G).^49^ All eleven assays were run according to the manufacturer’s instructions (Nordic Bioscience, Herlev, Denmark).

### Statistical analysis

Biomarker results were reported in accordance with the STROBE guideline (Supplemental File 1).^50^ Statistical analysis was done using R or the R2 Genomics analysis and visualization platform. Differences in concentration were tested using a Mann–Whitney *U*-test for which a *p*-value <0.05 was considered statistically significant. Elastic net Cox regression was performed with a 20x re-iteration random sampling procedure using 85% of our data to train the models, and 10-fold cross-validation to minimize the regression penalties (lambda). A sensitivity analysis with varying levels of lasso and ridge regression was also performed (alpha = 0, 0.25, 0.5, 0.75, and 1). Input variables were all stromal biomarkers, output was a Cox proportional hazard model for overall survival. Overall survival was further evaluated using Kaplan–Meier curves with a log-rank test and multivariate Cox regression, for both of which a p-value of < 0.05 was considered statistically significant. Cutoff was determined using ROC analysis based on survival status. Covariates were based on the SOURCE model for predicting overall survival.^51^ Missing stages or tumor locations were categorized separately as Tx, Nx or Mx. Exploratory subgroup analyses were based on histology (adenocarcinoma vs squamous cell carcinoma) and treatment intent (curative vs palliative). We used treatment intent instead of stage since M1 disease, based on supraclavicular lymph node metastases, can be cured, and M1 disease in patients who had metachronous metastases often had missing T and N-stage. Gene expression data was analyzed using Spearman correlations on average z-scores per sample.

## Results

### Stromal biomarkers are increased in patients with esophagogastric cancer compared to healthy controls

**Figure 2.**
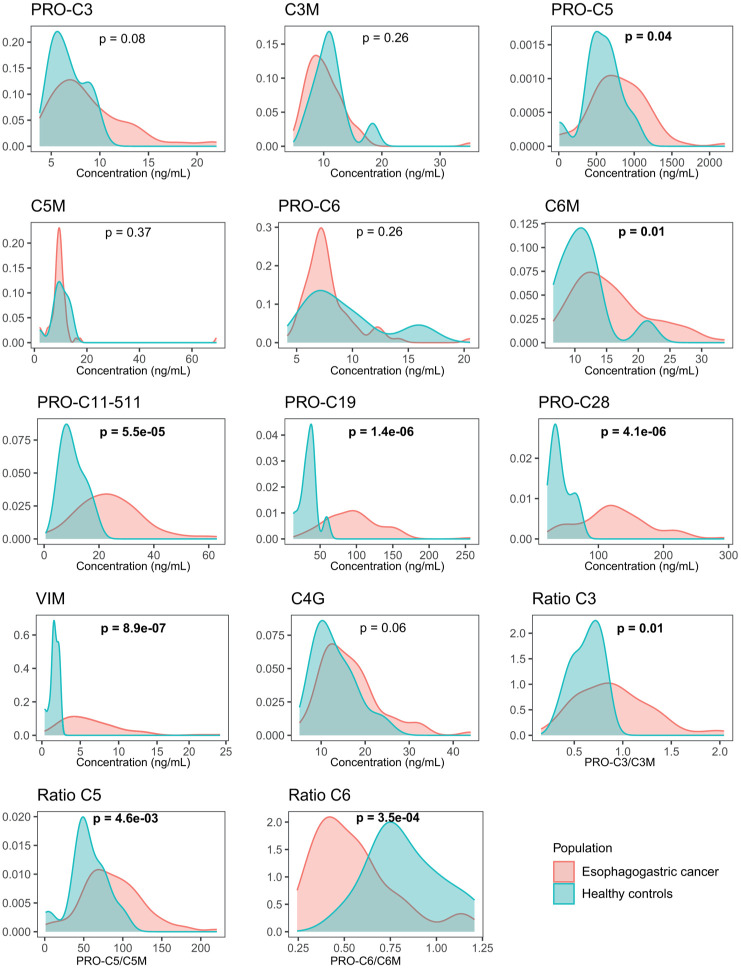
Distribution of stromal biomarkers between patients with EGC and healthy controls. Density plot per marker for patients with EGC and healthy controls. *p*-Values denote the Mann–Whitney *U*-test between populations. Statistically significant results are bolded. EGC, esophagogastric cancer.

To investigate whether stroma turnover is different in patients with EGC compared to healthy controls, we first evaluated the distribution of stromal biomarkers in these groups. The average concentration and 95% confidence interval per collagen fragment in patients with EGC are shown in Table 3. Formation fragments of collagen V, XI, and XXVII, and degradation fragments of collagen VI, XIX, and Vimentin were elevated in patients with EGC (Figure 2). The ratio of formation fragments of collagen III, V, and VI, divided by their corresponding degradation fragments, were statistically significantly different in patients with EGC compared to healthy controls. Thus, stroma turnover is increased in patients with EGC compared to healthy subjects.

**Table 3.** Average concentration in ng/mL and 95% confidence interval per collagen fragment.

Collagen marker	Mean concentration (ng/mL)	95% CI
PRO-C5	795.88	711.3–880.45
C6M	15.72	14.36–17.07
C5M	9.80	8.15–11.45
Ratio PRO-C5/C5M	86.89	78.01–95.76
PRO-C11-511	24.08	21.39–26.76
PRO-C28	124.13	111.78–136.49
PRO-C3	8.8	7.95–9.69
PRO-C6	7.85	7.30–8.40
VIM	6.70	5.76–7.66
Ratio PRO-C3/C3M	0.91	0.82–0.99
PRO-C19	95.72	86.91–104.52
Ratio PRO-C6/C6M	0.56	0.50–0.61
C3M	10.35	9.46–11.25
C4G	16.54	14.98–18.09

VIM, Vimentin.

### Serum levels of collagen V formation fragments associate with favorable prognosis in esophagogastric cancer

**Figure 3.**
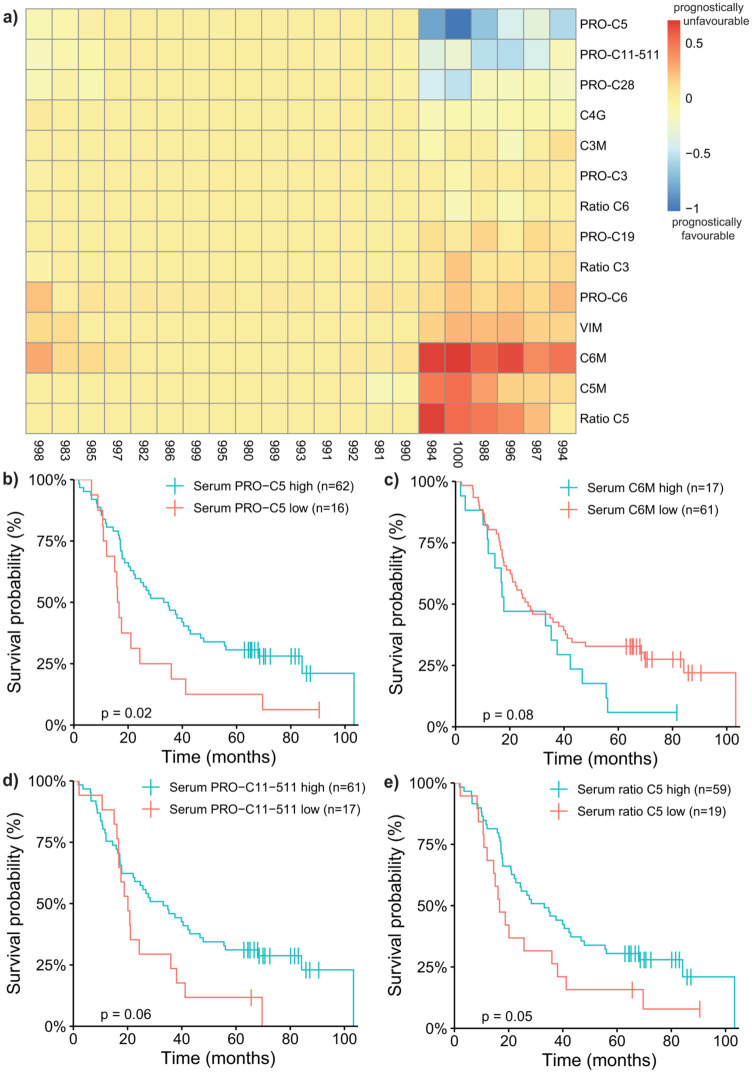
Relation between stromal biomarker in serum and overall survival. (a) Coefficients per marker in a 20x re-iteration random sampling procedure using 85% of our data to train the model and 10-fold cross-validation to minimize regression penalties. Blue denotes a negative coefficient while red denotes positive coefficients. Alpha was set at 0.25 for this particular elastic net Cox regression. (b) Kaplan–Meier survival analysis of patients with EGC with a high and low serum PRO-C5 (cut-off = 540.35 ng/mL based on ROC analysis). *p*-Value denotes the log-rank test. (c) Kaplan–Meier survival analysis of patients with EGC with a high and low serum C6M (cut-off = 19.40 ng/mL based on ROC analysis). *p*-Value denotes the log-rank test. (d) Kaplan–Meier survival analysis of patients with EGC with a high and low serum PRO-C11-511 (cut-off = 15.35 ng/mL based on ROC analysis). *p*-Value denotes the log-rank test. (e) Kaplan–Meier survival analysis of patients with EGC with a high and low serum ratio for collagen V (cut-off = 61.79 based on ROC analysis). *p*-Value denotes the log-rank test. EGC, esophagogastric cancer.

Next, we aimed to establish which turnover fragments were associated with overall survival in patients with EGC. For this, we applied an elastic net Cox regression, an unbiased approach to find out which, if any, stromal biomarker carried a prognostic signal. Median overall survival of all included patients was 26.2 months (IQR 16–64 months). Model generation was unstable, but higher levels of PRO-C5 and PRO-C11-511 consistently associated with lower hazard ratios (Figure 3(a)), while high levels of C6M and the ratio between PRO-C5 and C5M were prognostically unfavorable. We performed a sensitivity analysis varying the level of lasso and ridge regression, and these results remained robust (see Methods). Thus, higher concentrations of PRO-C5, the ratio between PRO-C5 and C5M, and C6M have prognostic potential in patients with EGC.

We next aimed to validate these results using Kaplan–Meier and Cox regression analyses. We used ROC analyses based on survival status to determine the optimal cutoff point per stromal biomarker. For PRO-C5, a threshold of 540.35 ng/mL was determined to best dichotomize high and low serum expression of PRO-C5. C6M was dichotomized using 19.4 ng/mL as a cutoff, PRO-C11-511 was dichotomized using 15.35 ng/mL, and the ratio PRO-C5/C5M had a threshold of 61.79. In patients with esophagogastric carcinoma, high serum levels of baseline PRO-C5 were prognostically favorable (Figure 3(b)). When correcting for age, gender, histological subtype, differentiation grade, TNM-status, and treatment, high levels of PRO-C5 remained independently associated with longer survival (HR 0.453, *p* = 0.05; Table 4). To further explore the prognostically favorable signal in patients with EGC, we plotted the distribution of PRO-C5 categorized by metastasis (Supplemental Figure 1). There was a trend toward PRO-C5 levels in patients with metastatic disease, supporting the prognostically favorable signal of high serum PRO-C5.

**Table 4.** Univariate and multivariate Cox regression for serum concentration of PRO-C5, PRO-C11-511, C6M and the ratio PRO-C5/C5M.

Cox regression analysis		Univariate		Multivariate PRO-C5		Multivariate PRO-C11-511		Multivariate C6M		Multivariate PRO-C5/C5M	
Variables		HR (95% CI)	*p*-Value	HR (95% CI)	*p*-Value	HR (95% CI)	*p*-Value	HR (95% CI)	*p*-Value	HR (95% CI)	*p*-Value
PRO-C5	High vs low*	0.503 (0.278–0.908)	**0.023**	0.453 (0.21–0.98)	**0.048**						
PRO-C11-511	High vs low*	0.562 (0.313–1.007)	0.053			0.821 (0.317–2.129)	0.685				
C6M	High vs low*	1.646 (0.341–1.082)	0.090					1.541 (0.265–1.590)	0.344		
Ratio PRO-C5/C5M	High vs low*	0.566 (0.322–0.997)	**0.049**							0.517 (0.243–1.101)	0.087
Age	Continuous	1.010 (0.979–1.043)	0.524	0.996 (0.960–1.034)	0.848	1.001 (0.964–1.040)	0.943	1.003 (0.967–1.040)	0.881	1.001 (0.965–1.038)	0.958
Gender	Male vs female*	0.678 (0.371–1.239)	0.206	0.583 (0.258–1.317)	0.194	0.503 (0.216–1.175)	0.113	0.471 (0.201–1.103)	0.083	0.542 (0.241–1.215)	0.137
Tumor type	- EAC*	Ref	Ref	Ref	Ref	Ref	Ref	Ref	Ref	Ref	Ref
	- ESC	0.862 (0.444–1.672)	0.661	0.637 (0.258–1.572)	0.328	0.609 (0.245–1.514)	0.286	0.524 (0.199–1.378)	0.190	0.607 (0.246–1.494)	0.277
	- EASC	0.000 (0.000–inf)	0.997	0.000 (0.000–inf)	0.997	0.000 (0.000–inf)	0.997	0.000 (0.000–inf)	0.997	0.000 (0.000–inf)	0.997
	- GAC	2.045 (0.864–4.844)	0.104	2.087 (0.653–6.671)	0.215	1.721 (0.551–5.372)	0.350	1.254 (0.360–4.373)	0.722	2.276 (0.689–7.515)	0.177
Differentiation grade	- 1*	Ref	Ref	Ref	Ref	Ref	Ref	Ref	Ref	Ref	Ref
	- 2	1.793 (0.615–5.230)	0.285	3.099 (0.663–14.48)	0.151	2.618 (0.602–11.38)	0.199	3.382 (0.750–15.26)	0.113	2.494 (0.561–11.08)	0.230
	- 3	3.059 (1.044–8.961)	**0.041**	4.756 (1.040–21.75)	**0.044**	4.269 (1.020–17.86)	**0.047**	4.849 (1.140–20.63)	**0.033**	3.839 (0.885–16.66)	0.072
cT	- 1*	Ref	Ref	Ref	Ref	Ref	Ref	Ref	Ref	Ref	Ref
	- 2	1.151 (0.143–9.255)	0.894	0.721 (0.069–7.547)	0.785	0.658 (0.056–7.772)	0.739	0.498 (0.050–4.980)	0.553	0.824 (0.080–8.506)	0.871
	- 3	1.984 (0.273–14.43)	0.499	1.320 (0.152–11.46)	0.802	1.134 (0.119–10.80)	0.913	0.873 (0.103–7.360)	0.900	1.489 (0.169–13.15)	0.720
	- 4	8.025 (0.819–78.63)	0.074	4.739 (0.385–58.30)	0.224	3.024 (0.255–35.83)	0.380	2.275 (0.196–26.42)	0.511	4.470 (0.369–54.16)	0.240
cN	- 0*	Ref	Ref	Ref	Ref	Ref	Ref	Ref	Ref	Ref	Ref
	- 1	1.613 (0.810–3.209)	0.173	1.161 (0.481–2.804)	0.740	1.314 (0.542–3.182)	0.545	1.404 (0.603–3.269)	0.431	1.070 (0.423–2.704)	0.887
	- 2	1.716 (0.802–3.672)	0.164	0.771 (0.274–2.111)	0.620	0.658 (0.236–1.831)	0.423	0.621 (0.228–1.694)	0.352	0.623 (0.223–1.746)	0.368
	- 3	2.704 (1.114–6.561)	**0.028**	1.266 (0.364–4.612)	0.720	1.243 (0.335–4.605)	0.745	1.165 (0.301–4.511)	0.825	1.263 (0.352–4.537)	0.720
cM	- 0*	Ref	Ref	Ref	Ref	Ref	Ref	Ref	Ref	Ref	Ref
	- 1	2.811 (1.612–4.901)	**<0.001**	1.152 (0.408–3.253)	0.790	1.319 (0.471–3.695)	0.598	1.231 (0.437–3.470)	0.694	1.110 (0.396–3.115)	0.843
Treatment	- Palliative chemo*	Ref	Ref	Ref	Ref	Ref	Ref	Ref	Ref	Ref	Ref
	- nCRT + surgery	0.369 (0.219–0.623)	**<0.001**	0.450 (0.1934–1.045)	0.063	0.444 (0.187–1.051)	0.065	0.447 (0.193–1.034)	0.060	0.405 (0.173–0.949)	**0.038**
	- dCRT	0.000 (0.000–inf)	0.995	0.000 (0.000–inf)	0.997	0.000 (0.000–inf)	0.997	0.000 (0.000–inf)	0.997	0.000 (0.000–inf)	0.997

Significant results are bolded.

* Denotes reference category.

Serum levels of C6M failed to validate the prognostically unfavorable signal found in the elastic net Cox regression in the Kaplan–Meier curve (Figure 3(c)) and in a Cox regression correcting for age, gender, histological subtype, differentiation grade, TNM-status, and treatment (HR 1.541, *p* = 0.34; Table 4). After correcting for age, gender, histological subtype, differentiation grade, TNM-status, and treatment, high serum levels of PRO-C11-511 (HR 0.821, *p* = 0.69; Figure 3(d) and Table 4), and a higher ratio of PRO-C5/C5M (HR 0.517, *p* = 0.09) did not replicate the prognostic signal found in the elastic net analysis (Figure 3(e) and Table 4). For PRO-C5, we performed an exploratory subgroup analysis based on histology and treatment intent (Supplemental Figure 2). In the subpopulation of patients with adenocarcinoma who were treated with curative intent, we again found that PRO-C5 was prognostically favorable. However, this trend disappeared in the metastatic subpopulation. Thus, increased serum concentration of the formation fragment of collagen V is independently prognostically favorable in patients with EGC.

### Serum formation fragment of collagen V is correlated with normal stroma

**Figure 4.**
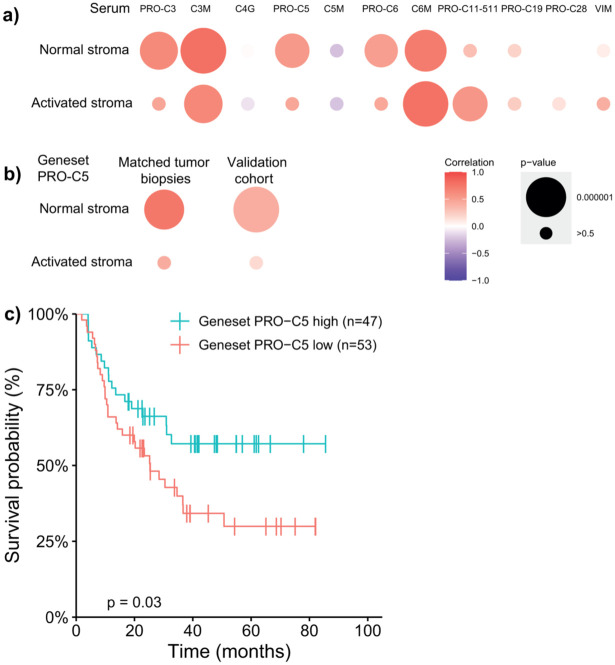
Correlation between serum marker and stromal subtypes explains their prognostic signal in two esophagogastric cancer datasets. (a) Correlation between the average *z*-score for the normal and activated stroma subtypes and serum concentration of stromal biomarker. (b) Correlation between average *z*-score for the normal and activated stroma subtypes and average *z*-score of PRO-C5 gene signature in the matched tumor and metastatic biopsies and a validation cohort of untreated tumor biopsies (GSE254942). (c) Kaplan–Meier survival analysis of patients with esophagogastric adenocarcinoma with a high and low average *z*-score for the PRO-C5 geneset (cutoff *z*-score = −0.02) in the validation cohort. *p*-Value denotes the log-rank test.

Distinct subtypes of the stroma have been shown to differentially associate with prognosis in patients with PDAC.^28^ A so-called “activated stroma” subtype is prognostically unfavorable, while “normal stroma” is prognostically favorable. We hypothesized that the opposing signals for different collagens found in the elastic net Cox regression may be related to these distinct stroma subtypes. We next used gene expression data to investigate this hypothesis. Gene expression data to determine the relative abundance of these stroma subtypes were available for 17 patients of the 78 patients in whom serum stromal biomarkers were determined. Similar to the complete dataset, the serum concentration of these 17 samples was higher compared to healthy controls (Supplemental Figure 3). Serum degradation fragments of collagen III and VI significantly correlated with both normal and activated stroma, while the formation fragments of collagen III, V, and VI only correlated significantly with a normal stroma signature, and the formation fragment of collagen XI only correlated with an activated stroma signature (Figure 4(a)). This corresponds to the prognostic signal of the biomarkers found in the elastic net Cox regression. Thus, we concluded that formation of collagen V is reflective of a normal stroma subtype, which is prognostically favorable in patients with esophagogastric carcinoma.

We next aimed to validate that formation of collagen V is reflective of a normal stroma subtype of desmoplasia in another dataset. We used a larger RNA-sequencing dataset of untreated tumor biopsies of patients with EGC treated in our hospital (validation cohort, *n* = 100).^52^ Serum stromal biomarker did not correlate consistently to mRNA of their respective proteins (Supplemental Figure 4 and Supplemental Table 1). Therefore, we first generated a gene signature of positively associated genes with the formation fragment of collagen V in the matched tumor biopsies (*p* < 0.01, no posthoc correction). This resulted in a gene signature of 230 genes (Supplemental Table 2). We similarly generated a gene signature of the prognostically unfavorable degradation fragment of collagen VI (Supplemental Table 3). We eliminated the overlapping 63 genes so we were left with a gene signature consisting of 167 genes (Supplemental Table 4). This eliminated those PRO-C5-associated genes that are less PRO-C5 specific and more related to general desmoplasia. When correlating this PRO-C5-specific gene signature to the stromal subtypes, we again found a significant positive correlation to normal stroma in both the matched tumor biopsies and the validation cohort of 100 biopsies (Figure 4(b)). An average *z*-score expression above −0.02 of this gene signature was also prognostically favorable in the validation cohort (HR 0.538 (0.301–0.963), *p* = 0.04), and this was independent of age, gender, differentiation grade, TNM-status, histological subtype, and treatment (HR = 0.471 (0.225–0.0984), *p* = 0.05; Figure 4(c)). Thus, the formation of collagen V reflects a distinct normal stroma subtype in patients with EGC that associates with favorable outcomes.

## Discussion

This study found that several stromal biomarkers are increased in patients with EGC, indicating that stromal turnover is different in patients with EGC compared with healthy subjects. The formation fragment of collagen V was found prognostically favorable, independent of clinical characteristics, and was reflective of a normal stromal subtype of desmoplasia.

Strikingly, altered turnover of different interstitial collagens has a variable association with prognosis in patients with EGC. In our population, we found that increased degradation of collagen VI was prognostically unfavorable. This is consistent with previous reports in colorectal cancer, biliary tract cancer, and PDAC, in which increased levels of collagen VI fragments were also prognostically unfavorable.^35,38,39^ However, the formation fragment of collagen V, and to a lesser extent, the formation fragment of collagen XI and the ratio between the formation and degradation fragment of collagen V, were consistently prognostically favorable. This is in stark contrast to previous findings in PDAC, where the formation fragment of collagen V was shown to be prognostically unfavorable.^34^ This opposing signal of serum levels of specific stromal biomarker in EGC has not been previously described, although several studies found that high serum levels of varying stromal biomarker and matrix metalloproteases were prognostically unfavorable across different cancer types.^14,32,36–39,53–55^

A recent gene expression study on collagens across solid tumor types also showed that collagen expression was generally associated with worse prognosis.^56^ Notably, *COL5A1* and *COL5A2* expression were associated with a higher hazard ratio across multiple cancer types, but not in gastric cancer. This study also showed that collagen expression was downregulated in prostate and ovarian cancer, indicating tumor-specific alterations in collagen turnover. For lung cancer, *COL8A* expression was significantly associated with a lower hazard ratio. Indeed, the tumor microenvironment is generally thought to differ between tumor types and even by metastasis site.^57^ Thus, tumor-specific altered collagen turnover could explain why formation of collagen V was found prognostically favorable in our patient population, in contrast to its role in PDAC. This is supported by the difference we found in our exploratory subgroup analysis based on histology: PRO-C5 was only prognostically favorable in patients with adenocarcinoma, although the limited sample size for subgroup analyses restricts our ability to draw firm conclusions.

Our study showed that the opposing signals of specific stromal biomarkers are related to distinct stromal subtypes, one of which is “normal stroma,” based on microdissected PDAC tumor tissue.^28^ Dysregulated stromal modeling is generally acknowledged as a hallmark of cancer progression and therapy resistance, but increasing evidence highlights a tumor-repressive role as well.^26,27,58,59^ This study supports an ambivalent role of desmoplasia in EGC, as in PDAC, and provides insight into a corresponding specific collagen composition. Since the formation fragment of collagen V is increased in patients with EGC compared to healthy controls, a “normal stroma” subtype of desmoplasia should not be confused with healthy stroma.

Several limitations should be acknowledged. External validation would ideally be performed in similar samples, requiring an independent cohort with available serum samples. We aimed to validate our results in two external cohorts through gene expression analysis. However, correlations between stromal biomarker and their respective mRNAs were inconsistent in our cohort, possibly due to posttranscriptional events.^60^ To circumvent this limitation, we generated a PRO-C5-specific gene signature, the high expression of which was independently associated with favorable prognosis in the validation cohort.

In addition, the relatively small sample size may have limited statistical power and reduced our ability to detect effects in subgroup analyses. The heterogeneity of our cohort with respect to tumor site and histology may also have introduced biological variability and residual confounding. Finally, the retrospective design precludes causal inference and is subject to selection and information bias. Therefore, our findings should be interpreted as hypothesis-generating and require validation in larger, prospective studies. Longitudinal studies are also needed to assess the dynamic changes in stromal turnover during disease progression and treatment.

Stratification of patients based on serum biomarker profiles may facilitate the design of clinical trials targeting stroma and support more personalized treatment approaches. Thus far, targeting tumor stroma as cancer therapy has yielded ambivalent results, likely reflecting the dual and context-dependent role of stroma in cancer.^12,61^ A noninvasive biomarker capable of identifying stromal subtypes most likely to benefit from targeting holds considerable therapeutic potential.

## Conclusion

In conclusion, the serum concentration of a formation fragment of collagen V is prognostically favorable in patients with EGC and is reflective of a normal subtype of desmoplasia.

## Supplemental Material

sj-docx-1-tam-10.1177_17588359261459109 – Supplemental material for Serum levels of the C-terminal pro-peptide of type V collagen have prognostic potential and are associated with a normal subtype of desmoplasia in a retrospective cohort of patients with esophagogastric cancer Supplemental material, sj-docx-1-tam-10.1177_17588359261459109 for Serum levels of the C-terminal pro-peptide of type V collagen have prognostic potential and are associated with a normal subtype of desmoplasia in a retrospective cohort of patients with esophagogastric cancer by Benthe H. Doeve, Neel I. Nissen, Vera Weeda, Mark P. G. Dings, Sybren L. Meijer, Hanneke W. M. van Laarhoven, Maarten F. Bijlsma and Nicholas Willumsen in Therapeutic Advances in Medical Oncology

sj-eps-2-tam-10.1177_17588359261459109 – Supplemental material for Serum levels of the C-terminal pro-peptide of type V collagen have prognostic potential and are associated with a normal subtype of desmoplasia in a retrospective cohort of patients with esophagogastric cancer Supplemental material, sj-eps-2-tam-10.1177_17588359261459109 for Serum levels of the C-terminal pro-peptide of type V collagen have prognostic potential and are associated with a normal subtype of desmoplasia in a retrospective cohort of patients with esophagogastric cancer by Benthe H. Doeve, Neel I. Nissen, Vera Weeda, Mark P. G. Dings, Sybren L. Meijer, Hanneke W. M. van Laarhoven, Maarten F. Bijlsma and Nicholas Willumsen in Therapeutic Advances in Medical Oncology

sj-eps-3-tam-10.1177_17588359261459109 – Supplemental material for Serum levels of the C-terminal pro-peptide of type V collagen have prognostic potential and are associated with a normal subtype of desmoplasia in a retrospective cohort of patients with esophagogastric cancer Supplemental material, sj-eps-3-tam-10.1177_17588359261459109 for Serum levels of the C-terminal pro-peptide of type V collagen have prognostic potential and are associated with a normal subtype of desmoplasia in a retrospective cohort of patients with esophagogastric cancer by Benthe H. Doeve, Neel I. Nissen, Vera Weeda, Mark P. G. Dings, Sybren L. Meijer, Hanneke W. M. van Laarhoven, Maarten F. Bijlsma and Nicholas Willumsen in Therapeutic Advances in Medical Oncology

sj-eps-4-tam-10.1177_17588359261459109 – Supplemental material for Serum levels of the C-terminal pro-peptide of type V collagen have prognostic potential and are associated with a normal subtype of desmoplasia in a retrospective cohort of patients with esophagogastric cancer Supplemental material, sj-eps-4-tam-10.1177_17588359261459109 for Serum levels of the C-terminal pro-peptide of type V collagen have prognostic potential and are associated with a normal subtype of desmoplasia in a retrospective cohort of patients with esophagogastric cancer by Benthe H. Doeve, Neel I. Nissen, Vera Weeda, Mark P. G. Dings, Sybren L. Meijer, Hanneke W. M. van Laarhoven, Maarten F. Bijlsma and Nicholas Willumsen in Therapeutic Advances in Medical Oncology

sj-eps-5-tam-10.1177_17588359261459109 – Supplemental material for Serum levels of the C-terminal pro-peptide of type V collagen have prognostic potential and are associated with a normal subtype of desmoplasia in a retrospective cohort of patients with esophagogastric cancer Supplemental material, sj-eps-5-tam-10.1177_17588359261459109 for Serum levels of the C-terminal pro-peptide of type V collagen have prognostic potential and are associated with a normal subtype of desmoplasia in a retrospective cohort of patients with esophagogastric cancer by Benthe H. Doeve, Neel I. Nissen, Vera Weeda, Mark P. G. Dings, Sybren L. Meijer, Hanneke W. M. van Laarhoven, Maarten F. Bijlsma and Nicholas Willumsen in Therapeutic Advances in Medical Oncology

sj-eps-6-tam-10.1177_17588359261459109 – Supplemental material for Serum levels of the C-terminal pro-peptide of type V collagen have prognostic potential and are associated with a normal subtype of desmoplasia in a retrospective cohort of patients with esophagogastric cancer Supplemental material, sj-eps-6-tam-10.1177_17588359261459109 for Serum levels of the C-terminal pro-peptide of type V collagen have prognostic potential and are associated with a normal subtype of desmoplasia in a retrospective cohort of patients with esophagogastric cancer by Benthe H. Doeve, Neel I. Nissen, Vera Weeda, Mark P. G. Dings, Sybren L. Meijer, Hanneke W. M. van Laarhoven, Maarten F. Bijlsma and Nicholas Willumsen in Therapeutic Advances in Medical Oncology

## Declarations

**Ethics approval and consent to participate:** This study was approved by the METC Amsterdam UMC (BiOES; METC 2013_241). All participants provided written informed consent.

**Consent for publication:** Not applicable.

**Competing interests:** M.F.B. has received research funding from Celgene, Frame Therapeutics, and Lead Pharma, and has acted as a consultant to Servier and Olympus. H.W.M.vL. Consultant or advisory role: BMS, Daiichi, Dragonfly, Eli Lilly, MSD, Nordic Pharma, Servier. Research funding and/or medication supply: Bayer, BMS, Celgene, Janssen, Incyte, Eli Lilly, MSD, Nordic Pharma, Philips, Roche, Servier. Speaker role: Astellas, Daiichi, Novartis. None of these parties was involved in the design of this study or drafting of the manuscript. N.I.N and N.W. are employed at Nordic Bioscience, involved in the discovery and development of serological biomarkers. All other authors have no competing interests to declare.

**Availability of data and materials:** The datasets analyzed during the current study are available as Supplemental Material and in the GEO repository under GSE254942 and on Figshare (https://figshare.com/s/2cf7cd28805715224594).^52^
